# Assessment of the preparedness of the Ugandan health care system to tackle more COVID-19 cases

**DOI:** 10.7189/jogh.10.020305

**Published:** 2020-12

**Authors:** Esther Ejiroghene Ajari, Daniel Ojilong

**Affiliations:** 1 College of Medicine, University of Ibadan, Ibadan, Oyo State, Nigeria; 2 The TriHealthon, Ibadan, Oyo State, Nigeria; 3 Center for Contemplative Science, University of Virginia, Charlottesville, Virginia, USA; 4 Faculty of Health Sciences, Busitema University, Mbale, Uganda; 5 Kumi District Local Government, Kumi, Uganda

Coronaviruses are human and animal pathogens causing mainly respiratory infections but their potential to cause serious diseases, even pandemics, with the right conditions have been predicted by several studies [1]. For example, in a 2017 briefing to the United States (US) president, the US military predicted the possibility of such an outbreak [2]. The Coronavirus Disease 2019 (COVID-19), a global pandemic caused by the novel SARS-CoV-2 virus, confirms the accuracy of such predictions. This disease which originated from Wuhan, China gave the rest of the world some window of opportunity to prepare to tackle a possible widespread outbreak. The Ugandan health care system, for example, had an 81-day pre-outbreak opportunity and still has a post-outbreak opportunity since it only has 413 confirmed COVID-19 cases, a relatively low figure, as at May 30, 2020 [3]. Did the system utilise the pre-outbreak opportunity? Is the system utilizing the post-outbreak opportunity? These are the questions this paper aims to address.

Uganda’s doctor-patient and nurse-patient ratio is approximately 1:25 000 and 1:11 000 respectively [4]. This is way below the WHO recommended doctor-patient ratio of 1:1000. Also, even though there is no official WHO recommended nurse-patient ratio, 1:11 000 is still inappropriate considering that most developed health care systems have a doctor:nurse ratio of 1:2-5. In addition, the latest report, on staffing levels in public health facilities in Uganda, pegged the figure at 72% [5]. This is below the acceptable standard. Also, the country’s health sector allocation is 8.9% of the national budget (for the 2019/20 fiscal year), down from 9.2% (in the 2018/19 fiscal year) [6]. This budget is 6.1% lower than the acceptable health sector allocation according to the Abuja Declaration of 2001.

In Uganda, there are 2 national referral hospitals, 4 specialized government hospitals, 14 regional referral hospitals and hundreds of lower rung hospitals. Of these, only one of the national referral hospitals, one of the specialized government hospitals and the 14 regional referral hospitals have been designated as COVID-19 treatment sites by the Ministry of Health as at May 30, 2020. Most of these facilities, especially the regional referral hospitals, are ill equipped to handle COVID-19 cases. Some experts project that 500 cases could completely overwhelm the health system [7]. Also, there are some reports that the country’s institutional quarantine system exposed quarantined individuals to further risk of infection [8]. The arguments, in support of these reports, are that these individuals mixed up and shared the same toilets and bathrooms, and some of them stayed in the quarantine facilities beyond the mandated 14-day quarantine period despite testing negative for the virus several times. This was, reportedly, due to non-compliance with quarantine guidelines, according to the Ministry of Health. Many of these individuals tested positive for the virus later on. The Uganda Human Rights Commission also reports receiving several complaints of lack of access to food and drugs from some of these individuals.

Furthermore, some studies report that 5%-7% of patients sick with COVID-19 requires intensive care unit (ICU) admission [9]. But a 2018 study reported the availability of only 55 functional ICU beds in 12 health care centers [10]. However, according to media reports, the recently refurbished and reopened Mulago Specialized Hospital increases to 102 the total number of ICU beds in the country. However, the hospital has no functional website for confirmation of this information, and the Ministry of Health has not confirmed this either. Thus, the 2018 study is a more reliable data source. Taking into account Uganda’s population size, of about 40 million people, this report shows that there are only 1.3 ICU bed per million population, implying a very limited access to intensive health care in the country. Moreover, 83% of these ICU facilities are located in Kampala city and 75% in private hospitals [10]. The uneven distribution of these facilities further impedes access to intensive health care. Thus, it is logical to assert that Uganda’s health care sytem might truly be overwhelmed if the number of confirmed COVID-19 cases increases. Furthermore, there is paucity of studies documenting the total number of ventilators in Uganda but it is likely that the number is either lower or the same with that of ICU beds.

Despite the above limitations in the health system, Uganda, so far, seems to have done comparatively better than its neighbors in keeping the number of confirmed cases down with no registered COVID-19 related deaths as at May 30, 2020 [3]. In addition, 17% of confirmed cases have been cured and discharged as at this date [3]. However, for two reasons, this situation report might not represent the true epidemiological picture of the outbreak in Uganda. First and foremost, as at May 30, 2020, only 96 825 COVID-19 confirmation tests have been done so far in the country [3]. This implies that lesser than 0.25% of Uganda’s population has been tested for the virus. Also, Uganda has the world’s youngest population (approximately 78% of Ugandans are below 30 years of age), and several studies have indicated that the Coronavirus infection in young people are mostly asymptomatic and with a low death incidence rate. Therefore, since, most COVID-19 cases in the country, has been among males, aged between 20-49 years, there is reason to suspect that the zero death incidence from the disease might not be due to the efficiency of the Ugandan health care system.

**Figure:**
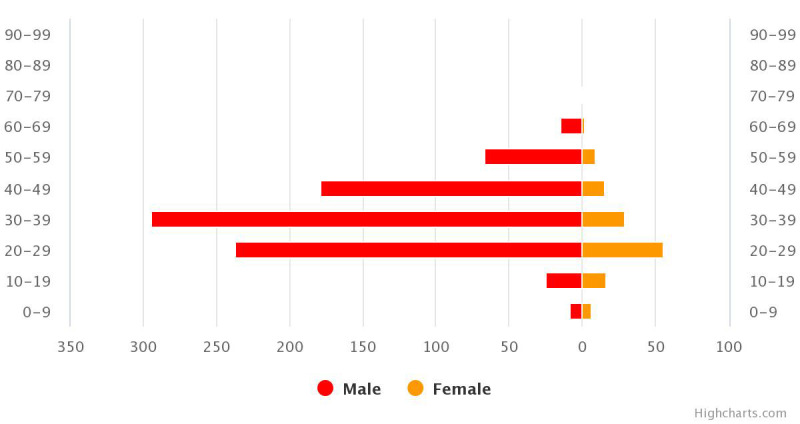
Photo: Graphic representation of the age and sex distribution of confirmed COVID-19 cases in Uganda. Retrieved July 10, 2020 (source: https://covid19.gou.go.ug/).

## CONCLUSION

Uganda did not completely neglect its window of opportunity for health care system strengthening, as necessitated by the pandemic. This is evident from the “no-death from COVID-19” situation in the country. However, it could have done better in upgrading several health care system development metrics in preparedness for the outbreak. This is, therefore, a call to Ugandan healthpolicy makers to heavily invest into the development of the country’s health sector and implement innovative and efficient strategies for managing more cases of the disease as they arise.
